# Exercise for Adolescents with Depressive Disorders: A Feasibility Study

**DOI:** 10.1155/2012/257472

**Published:** 2012-07-24

**Authors:** Richard R. Dopp, Ann J. Mooney, Roseanne Armitage, Cheryl King

**Affiliations:** ^1^Child and Adolescent Psychiatry Section, Department of Psychiatry, University of Michigan, 4250 Plymouth Road, Ann Arbor, MI 48109-2700, USA; ^2^Departments of Psychiatry and Psychology, University of Michigan, 4250 Plymouth Road, Ann Arbor, MI 48109-2700, USA; ^3^Sleep and Chronophysiology Laboratory, University of Michigan, 4250 Plymouth Road, Ann Arbor, MI 48109-2700, USA

## Abstract

*Objectives*. Adolescence is associated with increased depressive symptoms and decreased aerobic exercise, yet the relationship between exercise and clinical depression among adolescents requires further examination. This study assessed the feasibility of a 12-week intervention designed to increase exercise for adolescents with depressive disorders: Will a teenager with depression exercise? *Methods*. Participants were 13 adolescents with depression reporting low levels of aerobic exercise. They completed a 12-week intervention (15 supervised exercise sessions and 21 independent sessions). Exercise was measured through the aerobic exercise Questionnaire, actigraphy, and heart-rate monitoring. Depression was measured with the Children's Depression Rating Scale, Revised, and Quick Inventory of Depressive Symptomatology, Self-Report. *Results*. All participants who started the intervention completed the protocol, attending all supervised exercise sessions. Actigraphy verified 81% adherence to the protocol's independent sessions. Analysis of secondary outcomes showed a significant increase in exercise levels and a significant decrease in depression severity. Initially, ten participants were overweight or obese, and three were healthy weight. After 12 weeks of exercise, the number of participants in the healthy-weight category doubled. *Conclusions*. Adolescents suffering from depression can complete a rigorous protocol requiring structured increases in aerobic exercise. Participants showed significant increases in exercise, and significant decreases in depressive symptoms.

## 1. Introduction

Adolescent depression has long-term consequences and is often associated with additional depressive episodes in adulthood [1]. The presence of a major depressive disorder (MDD) in adolescents can negatively affect academic, social, and family functioning [2]. Evidence-based psychotherapies and medication are indicated for children and adolescents with moderate-to-severe depression [3], but greater than one-third of adolescents with depression do not show an adequate clinical response to antidepressant medications [4]. In the Treatment for Adolescents with Depression Study, the response rates after 12 weeks of treatment were 35% for placebo, 43% for cognitive behavioral therapy (CBT), 61% for fluoxetine, and 71% for combination treatment. However, the rates of full remission after 12 weeks of treatment were only 17% for placebo, 16% for CBT, 23% for fluoxetine, and 37% for combination treatment [5]. 

In addition to low rates of response and remission, there is concern regarding the safety of antidepressant medications. In 2005, the Food and Drug Administration issued a black-box warning stating that antidepressants may increase the risk for suicidal thinking and behavior in children and adolescents. This regulatory decision was based on a meta-analysis of 24 trials involving over 4,500 patients which indicated an increased risk of suicidality (risk ratio = 1.95; 95% CI = 1.28–2.98) during the first few months of initiating antidepressant medication treatment [6]. This warning has been associated with a decline in youth and young adults seeking treatment with antidepressant medications, prompting patients and parents to consider alternative, nonpharmacologic interventions [7].

One alternative treatment option currently under investigation is exercise. A randomized controlled trial (RCT) of older adults with mild-to-moderate depression compared supervised exercise, home-based exercise, sertraline, and pill placebo [8]. In this study, Blumenthal and colleagues demonstrated that exercise was as effective as medication for decreasing depressive symptoms. In a study of young adults with MDD, Dunn et al. assigned participants to one of five treatment conditions that varied by intensity (public health dose exercise, low-dose exercise, stretching control) and frequency (three or five times per week) [9]. In this DOSE study, all exercise sessions were conducted individually in a structured, supervised setting. After the intervention, an intent-to-treat analysis of participants' scores on the 17-item Hamilton Rating Scale for Depression (HRSD_17_) showed a reduction of 47% for participants in the public health dose exercise condition compared with 30% for the participants in the low dose condition and 29% for those in the stretching control condition. 

While vigorous exercise appears to be an effective treatment for depression in adults, a Cochrane Review suggests that the effect of exercise as treatment for depression in children and adolescents requires further investigation, as evidence-based research is lacking [10]. In nonclinical samples of adolescents, there is evidence to support that increases in leisure-time physical activity are associated with decreases in depressive symptoms [11]. Intervention research targeting health behavior change in the areas of physical activity and nutrition among adolescents in primary care has shown a positive impact on several health behaviors with the exception of vigorous physical activity [12]. Meanwhile, the strength of evidence in the use of exercise as treatment for depression in adults supports an emphasis on higher levels of aerobic activity [13]. Based upon these research findings showing that exercise is effective as treatment for adults with major depressive disorders, we chose to examine the feasibility of conducting similar research in adolescents with depression. We began by designing an intervention that required aerobic exercise three times per week. 

There are multiple challenges encountered when investigating exercise as treatment for depression in adolescents, including: (1) the adolescent desire for autonomy versus authoritarian demands of adults such as parents and medical professionals [14]; (2) the time commitments of adolescents with academic, social, and family demands; (3) expectations in society for quick-fix solutions to complicated medical illnesses like depression; (4) limited access to exercise facilities or safe environments for some youth [15]; and (5) sociocultural factors that may undermine some adolescents' efforts to change activity levels [16, 17]. There are additional challenges for the many adolescents who struggle with both depression and obesity. In children, depression is a risk factor for the development of obesity [18]. At the same time, obesity in childhood increases risk for later depression [19]. The factors that predispose individuals to depression, such as sleep disturbance and decreased activity levels, are some of the same factors that predispose individuals to obesity. Exercise may be more difficult to initiate and maintain for youth with both depression and weight issues.

A major concern in this area of research is the potential drop-out rate due to adolescents' many time commitments and potential loss of motivation. In reviewing the drop-out rates using exercise as treatment for adults with depression, Dunn's 2005 DOSE study reported that of the 80 randomized participants, 72 began exercise treatment and 53 completed 12 weeks, representing a drop-out rate of 34% [9]. In Blumenthal's 2007 SMILE study, supervised exercise and home-based exercise participants had drop-out rates of 20% and 6%, respectively [8]. Other research on exercise and depression in adults has proposed a combination of supervised and independent exercise sessions to reduce participant burden [20]. In designing our study protocol, we felt that requiring a combination of independent and supervised exercise sessions would encourage our adolescent participants to incorporate exercise into their lives. At the same time, we expected that adherence to, and completion of, our intervention protocol would still be potential challenges. 

The primary aim of this research study was to assess the feasibility of participant recruitment and retention in an exercise intervention for adolescents with depressive disorders. We hypothesized that there would be approximately a 25% drop-out rate during the study. Secondary outcomes included changes in levels of exercise and depression severity. Increases in exercise, as measured by actigraphy, heart rate monitoring during supervised sessions, and self-report, would suggest compliance with the protocol's requirements. Our corresponding secondary outcomes' hypotheses were that the frequency of exercise would increase and that depression severity would decrease for all participants.

## 2. Materials and Methods

### 2.1. Study Design

This study required adolescent participants with depressive disorders to complete a 12-week exercise intervention. The intervention included three supervised exercise sessions in the first week, two supervised exercise sessions in the second week, and one supervised exercise session in weeks 3 through 12. Participants were expected to complete one independent exercise session in week 2 and two independent exercise sessions in weeks 3 through 12. A final assessment was conducted three months following the conclusion of the exercise intervention. Beginning with the baseline appointment for the first participant and continuing through the three-month followup for the final participant, this study took 11 months to complete.

### 2.2. Participants

Institutional Review Board (IRB) approval was obtained to recruit participants through flyers distributed in local mental health clinics and high schools. Telephone screens were conducted with 25 adolescents to assess their levels of depression severity and physical activity, using the Quick Inventory of Depressive Symptomatology and the Physical Activity Questionnaire. If the adolescent reported symptoms of clinical depression and low levels of physical activity, he or she was invited to come in for a clinical baseline screening, at which the Children's Depression Rating Scale, Revised (CDRS-R) interview was conducted. A raw score of 36 or greater (T-Score of 61 or greater) on the CDRS-R was required for acceptance into the study. Screening took place over a four-month period to recruit 14 participants who met study eligibility. Eleven screened adolescents were excluded due to the following: elevated activity levels (*n* = 4), lack of willingness to exercise at required level (*n* = 2), failure to respond to research staff's attempts to set up initial interviews (*n* = 2), lack of depression (*n* = 1), unwillingness to ask parent for consent (*n* = 1), and diagnosis of bipolar disorder (*n* = 1). The adolescent with a prior diagnosis of bipolar disorder was excluded in order to maintain diagnostic homogeneity and due to a lack of published data regarding the effects of exercise on youth with bipolar disorder. 

Adolescent assent and parental consent were obtained from 14 adolescents and basic demographic information was collected. One participant sustained a major orthopedic injury prior to initiating the exercise intervention. Thus, the intervention phase was initiated with 13 participants (see Table 1).

These 13 participants included nine females (69%) and four males (31%) who ranged in age from 13 to 17 years (*M* = 15.2, SD = 2.4). Ethnicity and race were consistent with regional demographics: Caucasian (54%), African American (23%), biracial or multiethnic (15%), and Hispanic (8%). Eleven participants met criteria for MDD (85%), and two for Depressive Disorder, Not Otherwise Specified (15%). Four participants had MDD and a comorbid diagnosis: one with Attention-Deficit/Hyperactivity Disorder (ADHD); one with Anxiety Disorder, Not Otherwise Specified; two with both ADHD and Anxiety Disorder, Not Otherwise Specified.

At baseline, nine of the 13 participants (69%) were in psychotherapy and/or medication treatment for their depression. Seven participants (54%) were taking no psychotropic medications during the study. Five participants (38.5%) were on selective serotonin reuptake inhibitors (SSRI) at the beginning of the intervention (four on fluoxetine and one on sertraline) and continued on them throughout the intervention. Three of the five participants on SSRIs at baseline were also taking stimulants. During the intervention, two participants initiated psychotherapy, and one participant began taking an SSRI (sertraline) during the intervention at approximately the week 3 time point. Two unmedicated participants terminated their psychotherapy treatment for depression during the intervention, both citing an improvement in mood as the reason for termination.

### 2.3. Measures

#### 2.3.1. Physical Activity Questionnaire for Older Children (PAQ-A) [21]

The PAQ-A is a self-administered questionnaire which asks informants to recall activities which made them “sweat or make your legs feel tired, or games that make you breath hard” in the past seven days. A score of 1 on the PAQ-A is equivalent to no exercise in the last seven days. A score of 5 is equivalent to exercise greater than five times in the last seven days. The questionnaire assesses participation in specific activities and sports (e.g., walking, running, football, and basketball) as well as specific times (e.g., physical education classes, after school, evenings, and weekends). The PAQ-A was modified to include common types of physical activity in the study region. The PAQ-A has good internal consistency [22], and it has been shown to have convergent validity with concurrently used physical activity measures, including self-report questionnaires and motion sensors [23].

#### 2.3.2. Children's Depression Rating Scale, Revised (CDRS-R) [24]

The CDRS-R consists of a semistructured interview with strong concurrent validity, demonstrated by associations with other measures of child and adolescent depressive symptoms. It has shown high internal consistency (alpha = 0.85) in cross-sectional samples and good interrater reliability [25]. In this study, two staff participated in the administration of the CDRS-R at all time points and recorded their ratings separately. Interrater reliability proved to be high. Across 17 items with a possible total raw score ranging from 17 to 113, total ratings within five points of each other were considered as being in agreement. Inter-rater agreement was 89.7% over all three time points. After each clinical assessment, the clinicians compared ratings, discussed differences, and determined a consolidated score. Raw score ratings are 30–42 for mild depression, 43–57 for moderate, 58–72 for severe, and 73 or higher for very severe depression [26].

#### 2.3.3. Quick Inventory of Depressive Symptomatology (QIDS) [27]

The QIDS has highly acceptable psychometric properties which support the usefulness of this brief rating of depressive symptom severity in both clinical and research settings. Internal consistency is high for the self-report version, the QIDS-SR (Cronbach's alpha =  0.86). In a 2010 study of 140 adolescent outpatients, all versions of the QIDS (except the parent interview) were shown to have high reliability in use with adolescents [28]. Ratings on the QIDS-SR are 6–10 for mild depression, 11–15 for moderate, 16–20 for severe, and greater than 21 for very severe depression[27].

#### 2.3.4. Actigraphy (Actiwatch-LTM, Mini Mitter Co., Inc., Bend, OR) [29]

The actigraphs are worn on the wrist like a watch and electronically measure the number of movements that exceed 0.01 g (gravitation force per minute of recording). Data were downloaded from the actigraphs at the end of each recording period. Initial data analyses were conducted with on-board Mini-Mitter software.

#### 2.3.5. Body Mass Index (BMI)

The Center for Disease Control and Prevention promotes using BMI as a way of assessing body fat for children and adolescents. The CDC Teen BMI Calculator was used to determine categories for participants pre- and post-intervention. A healthy weight for a teen is indicated by being in the 5th to 84th percentile; one is considered overweight if in the 85th to the 94th percentile; and an adolescent is considered obese if he or she is in the 95th percentile or above [30].

### 2.4. Procedures

#### 2.4.1. Pre-Intervention Assessment

Baseline interviews were conducted with eligible adolescents and a parent or legal guardian. Self-report measures were completed by the participants, and clinical staff conducted the CDRS-R interview. Height and weight were measured at baseline, week 12, and three months post-intervention. The same scale (Scale-tronix Model #5002) was used for all weight measurements.

The participants wore an actigraph for one week to document activity levels and sleep schedules prior to the intervention. Participants were told to keep the actigraph on 24/7 for this week, as well as weeks 3 and 12. Those subsequent weeks of actigraphy provided us with data to confirm compliance with planned independent exercise sessions. This was conducted by reviewing the actogram and verifying the independent exercise sessions as reported by the participant.

#### 2.4.2. Intervention

Medical clearance was obtained from each participant's primary care physician prior to initiating exercise. In the first week of the exercise intervention, the adolescents came to the exercise laboratory three times for supervised sessions using a treadmill, an elliptical, and/or a stationary bike. These were individual sessions with only study staff present to collect self-report measures, monitor safety, and record heart rate. Participants were allowed to listen to music; many brought MP3 players or iPods, and a radio and CD player were provided for those without their own electronic devices. All pieces of exercise equipment had surfaces on which to put a book or magazine, so reading was allowed. One participant brought his laptop and watched online television programs during his exercise session. Listening to music was the most common activity.

Participants were encouraged to warm up slowly, to try all exercise equipment and develop their own preferences, and to increase the length of their workouts gradually. Each participant was asked to exercise for at least 30 minutes in the first supervised session and to exercise for 45 to 60 minutes by the second or third session and thereafter. Heart rates were recorded every 10 minutes using the Polar F55 BRO model heart rate monitor. Both the elliptical and the treadmill also had cardiogrip readings which helped the participants to be aware of their heart rate throughout the session.

Initially in this study, there was an expectation that working out on the available equipment (treadmill, elliptical, and stationary bike) would lead to an aerobic exercise session for participants. However, after six participants completed the 12-week intervention, it became apparent that the intensity of the supervised exercise varied greatly among the adolescents. It was determined that a target heart rate would be established for each participant. The algorithm used was 220 minus age, multiplied by 0.85. From that point forward, participants were told their target heart rate and were encouraged to hit that rate multiple times during their session. If that was accomplished easily within the first 10 to 15 minutes, they were encouraged to maintain that heart rate for as long as possible. The emphasis was on aerobic exercise. If participants hit and maintained their target heart rate during the first 40 minutes of the session, they were allowed to exercise using free weights for the last 10 minutes of their session. For participants who expressed interest in using the weights, this was used as an incentive to maintain aerobic levels in the first 40 minutes of their session.

In the second week, the participants had two supervised sessions at the exercise laboratory, and completed one exercise session independently. Suggestions were made for independent workouts, including using available equipment (at home, school, local gyms), jogging in safe neighborhoods or on tracks at school, or working out with exercise videos. Some participants made suggestions of their own: jumping rope, dancing with friends, and working out with exercise shows on cable television stations. 

In weeks 3 through 12 of the intervention, the staff reviewed with the participant what exercise sessions had been accomplished independently in the past week, and assisted the participant in planning the independent sessions for the following week. For each of the next 10 weeks, the participants had one supervised session at the exercise laboratory and exercised twice on their own. 

#### 2.4.3. Post-Intervention Assessment

Three months after the intervention, participants wore an actigraphy watch for one more week before returning for a final clinical assessment. They then had an interview during which the CDRS-R was administered and completed measures assessing levels of exercise and depression.

Participants received five $25 cash payments at baseline; weeks 4, 8, 12, and at the three-month post-intervention time point.

### 2.5. Statistical Analysis

Paired samples *t*-tests were conducted to compare baseline and post-intervention outcomes. Pearson's two-tailed correlations were used for the analysis of exercise level and depression severity. Cohen's *d* was computed to evaluate the effect size of changes in exercise and depression severity [31].

## 3. Results and Discussion

### 3.1. Results

#### 3.1.1. Feasibility

Every participant completed this exercise intervention. On average, it took 14.25 (SD = 3.1) weeks to complete the 15 supervised exercise sessions and the 21 independent sessions. Six participants completed the intervention in 12 consecutive weeks. Other participants took extra time to complete the study due to transportation problems, illness, vacations, and demands of high school academics. Adverse events which were reported to the IRB included minor orthopedic injuries and one suicide attempt. Additional negative life events also impacted time to completion, including temporary homelessness, parental separations (two participants), and a police arrest. 

Consistent with our aim to assess feasibility of participant retention, the adolescents were asked to give reasons for their participation so that we might better understand the motivational factors involved in getting adolescents with depression to exercise. The request was verbal, no menu of options was offered, and multiple answers were allowed. Reasons given by participants were to have structured exercise (*n* = 5), to lose weight (*n* = 5), for incentive money (*n* = 5), to fight their depression (*n* = 4), to be involved in research (*n* = 2), and to help other people (*n* = 1). In four cases, either parents or participants stated that they initiated participation in this research project as an alternative to antidepressant medication.

#### 3.1.2. Exercise Outcomes

**Figure 1:**
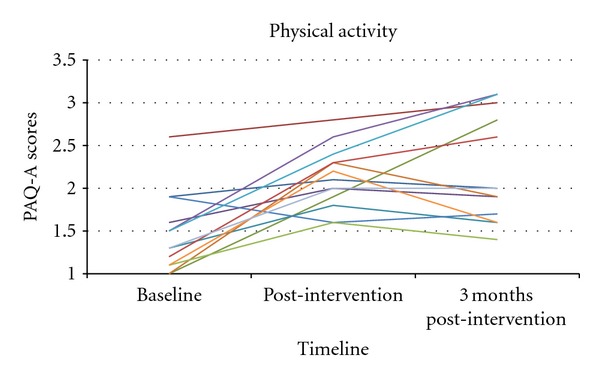
Adapted Physical Activity Questionnaire for Older Children scores at baseline, post-intervention, and three-month post-intervention time point.

In our study, heart rates were monitored every 10 minutes during the supervised exercise sessions, with averages ranging from 124 to 181 beats per minute. For participants exercising prior to the establishment of a heart rate goal (220 − age × 0.85), target heart rates were achieved in 39% of the supervised exercise sessions. After target heart rates were calculated and participants were encouraged to hit those rates, in the remaining supervised sessions of the study, they hit their targets in 79% of those sessions. As seen in Figure 1, scores on the adapted PAQ-A increased significantly during the intervention phase (*P* = 0.001). At baseline, the mean score on the adapted PAQ-A was 1.46 (SD = 0.48) with an increase to 2.10 (SD = 0.37) as measured during the final week of the intervention. The effect size based on Cohen's *d* = 1.6 was large, indicating robust effects. At the three-month post-intervention assessment, the mean score for the total sample on the adapted PAQ-A continued to rise to 2.21 (SD = 0.62).

Actigraphy recordings during weeks 3 and 12 were used to verify independent exercise sessions for those two weeks. Of the 52 expected independent physical activity sessions during those weeks, 42 sessions (81%) were verified by actigraphy. Of our 13 participants, one showed no actigraph data for both weeks 3 and 12; another had no actigraphy recorded for one of the two expected weeks. Absence of data was due to either technological malfunction of the actigraph or participant noncompliance (i.e., not wearing the actigraph as requested). Excluding the missing actigraphy, 91% of the expected independent exercise sessions were verified. 

#### 3.1.3. Depression Outcomes

**Figure 2:**
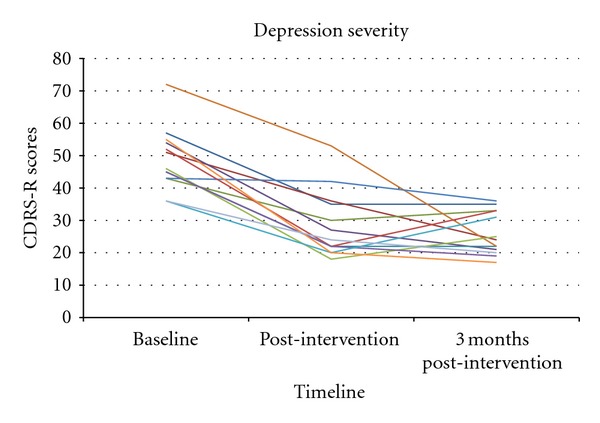
Children's Depression Rating Scale scores at baseline, post-intervention and three-month post-intervention time point.

The depression outcome measure was change in the CDRS-R score from baseline to completion of the exercise intervention, as seen in Figure 2. Depressive symptoms decreased significantly during the period of supported exercise (*P* < 0.001).

The mean score on the CDRS-R for these 13 participants was 48.9 (SD = 9.7) at baseline, and 28.5 (SD = 10.4) post-intervention, a decrease of more than 20 points. The effect size associated with depression improvement was also large, indicating robust effects on depression (Cohen's *d* = 2.0). Remission (CDRS-R < 28) was achieved by 62% of participants at the conclusion of the intervention. Three months after their final supervised exercise session, nine of the 13 participants had either maintained the same CDRS-R score or had a further decrease in depressive symptoms; mean score three months post-intervention was 25.9 (SD = 6.5). In this study, the mean score on the QIDS-SR was 10.2 (SD = 4.5) pre-intervention, 7.8 (SD = 5.5) post-intervention, and 6.2 (SD = 2.2) at the follow-up assessment three months after the intervention. 

#### 3.1.4. Body Mass Index

At baseline, five participants (38.5%) were obese, five (38.5%) were overweight, and only three (23%) were at a healthy weight. Between the baseline assessment and the conclusion of the exercise intervention, eight participants had a reduction in BMI while five participants had an increase in BMI. Following the intervention, three of the participants who had been in the overweight category were in the healthy-weight category. Of the three participants who moved to the healthy weight category, their mean BMI scores changed from 24.4 (SD = 1.4) pre-intervention to 23.6 (SD = 1.4) at 12 weeks. 

Baseline BMI values (*r* = −0.66, *P* = 0.03) and post-intervention BMI values (*r* = −0.75, *P* < 0.01) were negatively correlated with average heart rates recorded during the supervised exercise sessions. Higher heart rates were positively correlated with change in BMI (*r* = 0.75, *P* < 0.01); the overweight adolescents who reached higher heart rates showed greater reductions in BMI.

### 3.2. Discussion

In this exercise intervention, adolescents with clinically significant depression were capable of completing a rigorous protocol requiring structured increases in exercise. Despite adopting a combination of supervised and independent exercise sessions, we expected that compliance with, and completion of, our intervention protocol would still be major challenges. However, 100% of these adolescent participants completed the 15 individual, supervised sessions required in our intervention protocol. Furthermore, adolescents reported ongoing exercise at the three-month post-intervention assessment, with six participants reporting an increase in exercise beyond the level reported at the conclusion of the intervention. This suggests the acceptability of incorporating exercise into their daily lives. 

Given that our participants' compliance exceeded expectations, we reviewed the feasibility challenges we had expected to encounter and sought to identify which parameters of our study may have positively impacted those compliance and completion rates. First, the adolescents' independence was encouraged throughout our study. While parents or guardians were required to be involved in the consent process, during the period of exercise intervention all research staff communicated directly with the adolescent whenever possible rather than with the parents or guardians. Each week, participants reported to staff how they had met the independent exercise requirements, and goals for the following week were discussed. If parents asked if they should supervise or encourage the participant's independent sessions, research staff told them they were “off duty” for the independent sessions and that the teen would deal directly with the research staff regarding completion and reporting of the independent exercise. This helped to avoid having research expectations become a control issue or power struggle between adolescents and parents. The adolescents came up with creative ways of completing their independent sessions and were often proud of their exercise. 

Second, having the exercise facilities in the same building as the outpatient psychiatric clinic building made the supervised sessions convenient for those participants who were also patients at that clinic. Third, research staff were extremely flexible in accommodating appointment hours outside of school, offering times during afternoons, evenings and weekends. To implement exercise as treatment for adolescents with depression in real world clinical practice, attention must be paid to issues such as encouraging adolescent autonomy, keeping appointment options flexible and suited to adolescents' demanding schedules, and establishing equitable access to facilities (e.g., onsite equipment, discounted or free passes to local gyms). Lastly, our experience is consistent with other researchers who report that compliance with the protocol is developed and maintained through an excellent therapeutic alliance between research staff and participants [32].

In addition to examining the factors that made this research study feasible, we also examined our secondary outcomes to understand the changes in exercise levels and depression severity. These 13 participants significantly increased their aerobic exercise levels as evidenced by the monitoring of heart rates in the weekly supervised exercise sessions; heart rates were even higher when specific targets were set for the participants to achieve. Actigraphy was recorded during weeks 3 and 12, verifying 81% adherence to the protocol's independent exercise sessions. Actigraphs were downloaded following the week of recording and did not provide any feedback to the participants. However, in future studies, independent exercise sessions and other increases in exercise will be verified using data from pedometers to provide more consistent monitoring of activity levels. The use of pedometers may help to reinforce positive health behavior by providing real-time feedback regarding one's level of activity. Furthermore, adolescents wearing pedometers could make a connection between their level of activity and their mood state. 

Changes in depression severity were also assessed as a secondary outcome. Recognizing that previous research investigating exercise for adolescents with depression had been inconclusive in part due to a lack of repeated measures of depression severity [13], the design of this feasibility study included both clinician-rated and self-report measures of depression at multiple time points. All participants had reductions in their depression on both self-report (QIDS-SR) and the clinician-administered measure (CDRS-R). Of note, there was no control group in this research study. There is no way to confirm that the changes in depression were directly related to the exercise intervention. At the same time, several of these adolescents with depression had been engaged in psychotherapy and medication treatment for many months and in some cases for several years. Some of the adolescent participants reported that the exercise intervention had been the reason for their reduction in depressive symptoms.

Although weight was not considered in our inclusion criteria, 10 of our 13 participants were either overweight or obese at baseline. Also, five participants reported that losing weight was a motivating factor for their participation in this research. In this study, we found it feasible to both recruit and retain participants in all weight categories. For most participants, weight and BMI did not show significant change. However, several participants reported changes in how their clothing fit and felt positively about their change in physical appearance irrespective of a change in weight or BMI. 

Alternative and augmenting treatment options, such as exercise, should be explored for all adolescents with depression, especially those who are overweight or obese. Obesity alone predisposes individuals to coronary heart disease, type 2 diabetes, certain cancers, hypertension, dyslipidemia, stroke, sleep apnea, respiratory problems, osteoarthritis, gynecologic problems, and liver and gallbladder disease. For adolescents dealing with depression, a positive health behavior change such as increasing exercise could prove to promote self-management of depressive symptoms while also serving to prevent nonpsychiatric medical comorbidity.

In addition to including adolescents with a range of body mass indices, we allowed participants to continue concurrent treatment with medication and/or psychotherapy. By including participants with varying symptom profiles and at various stages of treatment, the results of this study suggest that it is feasible to consider exercise as a primary treatment for certain patients and as an adjunctive treatment for others. Of these 13 adolescents with depression, eight participants (62%) achieved remission of their depressive symptoms following the exercise intervention. However, one participant, with a minimal decrease in depression, showed a greater treatment response after fluoxetine was initiated during the three-month follow-up period. For this individual, exercise alone did not appear to be the most effective treatment, supporting the ongoing need to consider multiple modalities of treatment. Interestingly, the five participants who were on antidepressant medications at the start of the exercise intervention had impressive reductions in CDRS-R scores, with three achieving remission. This suggests that the exercise may have been a useful adjunctive treatment to pharmacotherapy in these individuals. Future studies, with efficacy as the primary focus, should consider starting with untreated adolescents with depression to more clearly delineate the antidepressant effects of exercise from those of medication and psychotherapy. 

Research on exercise and depression in adolescents will also need to address the challenging issues associated with control conditions, considering both scientific and safety questions. What is an appropriate control or “sham” condition in exercise research? Is it ethical to enroll adolescents with depressive disorders in a research protocol that includes randomization to conditions with unproven effectiveness in treating depression (i.e., exercise or placebo)? Previous researchers have explored several strategies to address these questions. Some have chosen to use stretching as a control condition which showed antidepressant effects similar to those of low-dose exercise [9]. In Blumenthal's SMILE study comparing exercise and medication, one of the conditions was a placebo control [8]. It has been suggested that the prospect of direct benefit to the participant is present even in RCTs with placebo conditions, as “the randomized design offers a prospect of active treatment to each participant” [33]. Many adolescents show a response to placebo [5] and a consensus statement of depression researchers stressed that placebo should not be considered as an absence of treatment [34]. Furthermore, a meta-analysis of studies in depression concluded that up to one in five participants in a wait-list control may experience remission of depression [35]. In combination with these published articles, the results of our study, which included both medicated and unmedicated participants, suggest that research investigating exercise as treatment for depression in adolescents is feasible and that randomized controlled trial designs should be considered.

#### 3.2.1. Study Limitations and Strengths

Methodological limitations included the lack of an objective measure of acceptability, the lack of a control group, and the lack of blinding for clinical staff administering assessments. We also acknowledge that these participants were self-selected and that these results may not be generalizable to all adolescents with depressive disorders. In addition, these data from 13 participants lack statistical power for additional analyses that might further explore gender differences and other questions about the relationships among exercise, depression, and obesity. 

In this study, exercise was measured via self-report, through heart rate monitoring, and with intermittent actigraphy. Despite the periodic use of actigraphy, there was no objective measure of exercise for the majority of the independent exercise sessions. In future research, we will strengthen this element of our investigation with the use of pedometers throughout the study period, which can assess more continuous compliance with the protocol as well the intensity of exercise. 

## 4. Conclusions

Our primary aim was to assess the feasibility of recruiting and retaining adolescents with depression in an exercise intervention. In short, can you get a teenager with depression to exercise? These adolescents with clinically significant depression completed a rigorous research protocol examining the role of exercise in the treatment of depression. The feasibility of research in this area is supported by the 100% completion of 15 supervised exercise sessions, and the strong adherence to the protocol's independent sessions. During supervised sessions, the documented heart rates confirmed the participants' ability to achieve elevated levels of aerobic activity. Examination of secondary outcome measures showed that these adolescents had a significant increase in their exercise levels and a significant decrease in their depressive symptoms. 

Ongoing research on depressive disorders and exercise is urgently needed to address the emerging epidemics of adolescent depression and obesity. Once the effectiveness of exercise is established as a treatment for depression in adolescents, the research must then focus on strategies to facilitate the implementation of this knowledge in the adolescents' world (e.g., psychiatric settings, school environment), and making the research results a catalyst for real behavioral change. If regular exercise can be proven to decrease depressive symptoms in adolescents, the information can and should impact clinical practice, encouraging the inclusion of exercise as part of the treatment plan. The ultimate goal is to use evidence-based research on exercise to influence the course of depressive illness during this vulnerable developmental stage.

## Figures and Tables

**Table 1:** Demographic characteristics of participants at baseline.

Baseline demographics	(*N* = 13)
Age	13–17 years, *M* = 15.2, SD = (1.4)
Sex	
Males	4
Females	9
Race	
Caucasian	7 (54%)
African American	3 (23%)
Biracial/MultiEthnic	2 (15%)
Hispanic	1 (8%)
Medical diagnoses	
Major depressive disorder	11 (85%)
Depressive disorder NOS	2 (15%)
Co-morbid diagnoses	
Attention deficit/hyperactivity disorder	2 (15%)
Anxiety disorder, NOS	2 (15%)
Medications	
None	7 (54%)
Selective serotonin reuptake inhibitor	5 (38.5%)
Fluoxetine	4 (30%)
Sertraline	1 (8%)
Stimulants (also on SSRIs)	3 (23%)
Body mass index—CDC category	
Healthy weight	2 (23%)
Overweight	5 (38.5%)
Obese	5 (38.5%)

## References

[1] Kovacs M (1996). Presentation and course of major depressive disorder during childhood and later years of the life span. *Journal of the American Academy of Child and Adolescent Psychiatry*.

[2] Puig-Antich J, Kaufman J, Ryan ND (1993). The psychosocial functioning and family environment of depressed adolescents. *Journal of the American Academy of Child and Adolescent Psychiatry*.

[3] Birmaher B, Brent D, Bernet W (2007). Practice parameter for the assessment and treatment of children and adolescents with depressive disorders. *Journal of the American Academy of Child and Adolescent Psychiatry*.

[4] Brent D, Emslie G, Clarke G (2008). Switching to another SSRI or to venlafaxine with or without cognitive behavioral therapy for adolescents with SSRI-resistant depression: the TORDIA randomized controlled trial. *Journal of the American Medical Association*.

[5] March J, Silva S, Vitiello B (2006). The Treatment for Adolescents with Depression Study (TADS): methods and message at 12 weeks. *Journal of the American Academy of Child and Adolescent Psychiatry*.

[6] Hammad TA, Laughren T, Racoosin J (2006). Suicidality in pediatric patients treated with antidepressant drugs. *Archives of General Psychiatry*.

[7] Libby AM, Brent DA, Morrato EH, Orton HD, Allen R, Valuck RJ (2007). Decline in treatment of pediatric depression after FDA advisory on risk of suicidality with SSRIs. *American Journal of Psychiatry*.

[8] Blumenthal JA, Babyak MA, Doraiswamy PM (2007). Exercise and pharmacotherapy in the treatment of major depressive disorder. *Psychosomatic Medicine*.

[9] Dunn AL, Trivedi MH, Kampert JB, Clark CG, Chambliss HO (2005). Exercise treatment for depression: efficacy and dose response. *American Journal of Preventive Medicine*.

[10] Larun L, Nordheim LV, Ekeland E, Hagen KB, Heian F (2006). Exercise in prevention and treatment of anxiety and depression among children and young people. *Cochrane Database of Systematic Reviews*.

[11] Motl RW, Birnbaum AS, Kubik MY, Dishman RK (2004). Naturally occurring changes in physical activity are inversely related to depressive symptoms during early adolescence. *Psychosomatic Medicine*.

[12] Patrick K, Sallis JF, Prochaska JJ (2001). A multicomponent program for nutrition and physical activity change in primary care: PACE^+^ for adolescents. *Archives of Pediatrics and Adolescent Medicine*.

[13] Dunn AL, Weintraub P (2008). Exercise in the prevention and treatment of adolescent depression: a promising but little researched intervention. *American Journal of Lifestyle Medicine*.

[14] Sawyer SM, Aroni RA (2005). Self-management in adolescents with chronic illness. What does it mean and how can it be achieved?. *Medical Journal of Australia*.

[15] Romero AJ (2005). Low-income neighborhood barriers and resources for adolescents’ physical activity. *Journal of Adolescent Health*.

[16] Robbins LB, Pender NJ, Kazanis AS (2003). Barriers to physical activity perceived by adolescent girls. *Journal of Midwifery and Women’s Health*.

[17] Henderson KA, Ainsworth BE (2003). A synthesis of perceptions about physical activity among older African American and American Indian women. *American Journal of Public Health*.

[18] Duarte CS, Sourander A, Nikolakaros G (2010). Child mental health problems and obesity in early adulthood. *Journal of Pediatrics*.

[19] Mustillo S, Worthman C, Erkanli A, Keeler G, Angold A, Costello EJ (2003). Obesity and psychiatric disorder: developmental trajectories. *Pediatrics*.

[20] Trivedi MH, Greer TL, Grannemann BD, Chambliss HO, Jordan AN (2006). Exercise as an augmentation strategy for treatment of major depression. *Journal of Psychiatric Practice*.

[21] Crocker PRE, Bailey DA, Faulkner RA, Kowalski KC, Mcgrath R (1997). Measuring general levels of physical activity: preliminary evidence for the physical activity questionnaire for older children. *Medicine and Science in Sports and Exercise*.

[22] Janz KF, Lutuchy EM, Wenthe P, Levy SM (2008). Measuring activity in children and adolescents using self-report: PAQ-C and PAQ-A. *Medicine and Science in Sports and Exercise*.

[23] Kowalski KC, Crocker PRE, Kowalski NP (1997). Convergent validity of the physical activity questionnaire for adolescents. *Pediatric Exercise Science*.

[24] Poznanski EO, Mokros H (1996). *Children’s depression rating scale revised (CDRS-R)*.

[25] Jain S, Carmody TJ, Trivedi MH (2007). A psychometric evaluation of the CDRS and MADRS in assessing depressive symptoms in children. *Journal of the American Academy of Child and Adolescent Psychiatry*.

[26] Poznanski EO, Mokros H Children's depression rating scale revised (CDRS-R)—manual (w-242). http://portal.wpspublish.com/portal/page?_pageid=53,69676&_dad=portal&_schema=PORTAL.

[27] Rush AJ (2003). The 16-item Quick Inventory of Depressive Symptomatology (QIDS), clinician rating (QIDS-C), and self-report (QIDS-SR): a psychometric evalution in patients with chronic major depression. *Biological Psychiatry*.

[28] Bernstein IH, Rush AJ, Trivedi MH (2010). Psychometric properties of the Quick Inventory of Depressive Symptomatology in adolescents. *International Journal of Methods in Psychiatric Research*.

[29] Actigraphy http://www.healthcare.philips.com/main/homehealth/sleep/actiwatch/default.wpd.

[30] CDC Teen BMI Calculator http://www.cdc.gov/healthyweight/assessing/bmi/childrens_bmi/about_childrens_bmi.html.

[31] Cohen J (1988). *Statistical Power Analysis for the Behavioral Sciences*.

[32] Hughes CW, Emslie GJ, Crismon ML (2007). Texas children’s medication algorithm project: update from Texas consensus conference panel on medication treatment of childhood major depressive disorder. *Journal of the American Academy of Child and Adolescent Psychiatry*.

[33] Vitiello B (2003). Ethical considerations in psychopharmacological research involving children and adolescents. *Psychopharmacology*.

[34] Charney DS, Nemeroff CB, Lewis L (2002). National depressive and manic-depressive association consensus statement on the use of placebo in clinical trials of mood disorders. *Archives of General Psychiatry*.

[35] Posternak MA, Miller I (2001). Untreated short-term course of major depression: a meta-analysis of outcomes from studies using wait-list control groups. *Journal of Affective Disorders*.

